# Diagnostic test accuracy of glutamate dehydrogenase for Clostridium difficile: Systematic review and meta-analysis

**DOI:** 10.1038/srep29754

**Published:** 2016-07-15

**Authors:** Jun Arimoto, Nobuyuki Horita, Shingo Kato, Akiko Fuyuki, Takuma Higurashi, Hidenori Ohkubo, Hiroki Endo, Nonaka Takashi, Takeshi Kaneko, Atsushi Nakajima

**Affiliations:** 1 Department of Gastroenterology and Hepatology, Yokohama City University School of Medicine, Yokohama, Japan; 2 Department of Pulmonology, Yokohama City University Graduate School of Medicine, Yokohama, Japan

## Abstract

We performed this systematic review and meta-analysis to assess the diagnostic accuracy of detecting glutamate dehydrogenase (GDH) for Clostridium difficile infection (CDI) based on the hierarchical model. Two investigators electrically searched four databases. Reference tests were stool cell cytotoxicity neutralization assay (CCNA) and stool toxigenic culture (TC). To assess the overall accuracy, we calculated the diagnostic odds ratio (DOR) using a DerSimonian-Laird random-model and area the under hierarchical summary receiver operating characteristics (AUC) using Holling’s proportional hazard models. The summary estimate of the sensitivity and the specificity were obtained using the bivariate model. According to 42 reports consisting of 3055 reference positive comparisons, and 26188 reference negative comparisons, the DOR was 115 (95%CI: 77–172, I^2^ = 12.0%) and the AUC was 0.970 (95%CI: 0.958–0.982). The summary estimate of sensitivity and specificity were 0.911 (95%CI: 0.871–0.940) and 0.912 (95%CI: 0.892–0.928). The positive and negative likelihood ratios were 10.4 (95%CI 8.4–12.7) and 0.098 (95%CI 0.066–0.142), respectively. Detecting GDH for the diagnosis of CDI had both high sensitivity and specificity. Considering its low cost and prevalence, it is appropriate for a screening test for CDI.

Clostridium difficile is an anaerobic, spore-forming Gram-positive bacillus that is capable of causing diarrhea mediated by the production of C. difficile toxins A and B^1^. C. difficile infection (CDI) accounts for 15% to 25% of antibiotic-associated diarrhea^2^. The two serious risk factors of CDI are exposure to antibiotics exposure to the organism, usually during a hospital stay. Others factors are older age, gastrointestinal tract surgery, and anti-acid medications including proton-pump inhibitors^3^,^4^. The severity of CDI ranges from very mild to toxic megacolon with septic shock. Metronidazole and vancomycin are the most frequently used first-line antibiotics to treat CDI. Fecal microbiota transplantation has recently been proposed as alternative treatment^5^,^6^. However, patients who do not respond to these medications may require intensive care or colectomy. According to surveillance, mortality from CDI is approximately 5.7%^7^.

The initial step in proper treatment of CDI is quick and accurate diagnosis of CDI. However, none of the existing C. difficile examinations is perfect in view of accuracy, cost, and incubation time^8^,^9^,^10^,^11^. Nucleic acid amplification tests (NAATs) such as polymerase chain reaction and loop-mediated isothermal amplification provide quick and accurate diagnosis^12^,^13^,^14^, albeit a high cost. Though expensive, single-step diagnosis strategies utilizing only a NAAT is the simplest diagnosis strategy^8^. Multiple-step diagnosis is another strategy for which low cost exam, namely glutamate dehydrogenase (GDH) assay, is used as the first-step tool, followed by NAATs or by toxin tests only for specimens with positive result in the first test^8^. Detecting GDH seems a reasonable screening tool because this non-expensive and non-time-consuming test is sensitive^15^.

Since the last decade, an increasing number of observational studies concerning GDH assay accuracy for C. difficile detection have been reported^15^. The current understanding is that single-step GDH assay could not confirm the CDI. Nonetheless, evaluation of the single-step GDH assay is necessary for some reasons. Single-step GDH assay negative usually warrants CDI negative. In addition, we had to know the diagnostic test accuracy of single-step GDH assay to design two-step and three-step GDH assays. Shetty *et al*. reported a systematic review concerning this topic in 2011^15^. However, due to considerable heterogeneity among studies, their study mainly focused on describing the summary receiver operating characteristic (SROC) curve and avoided presenting accurate pooled sensitivity and specificity. They avoided it because univariate meta-analysis leads to gross underestimates of sensitivity and specificity when the diagnostic test performance differs owing to local conditions^15^. Even though GDH is commonly accepted as a screening tool for CDI, no published meta-analysis has provided straightforward summary estimates of sensitivity and specificity of GHD to diagnose CDI. The recent meta-analysis methodology for diagnostic test accuracy strongly recommends use of a hierarchical model, which enables us appropriately deal with the tradeoff between sensitivity and specificity caused by the threshold effect^16^,^17^,^18^,^19^. In addition, many original studies have been published concerning GDH since the review by Shetty *et al*. was published. Thus, we believe an updated systematic review and meta-analysis using a hierarchical model is required to reveal how accurate the GDH assay is in diagnosing CDI.

## Methods

### Study registration

The protocol has been registered with the international prospective register of systematic reviews (PROSPERO) as number CRD42016032760^20^. This study protocol follows the Preferred Reporting Items for Systematic Reviews and Meta-Analyses (PRISMA) statement and the Cochrane Handbook for Diagnostic Test Accuracy Reviews^16^,^21^. Institutional review board approval and patient consent were waivered because of the review nature of this study.

### Eligibility criteria

#### Type of studies

We had planned to include both two-gate cohort studies and one-gate case-control studies. However, we eventually found no case-control study. We included a study with sufficient data to estimate the sensitivity and the specificity of GDH assay for CDI using PCR as reference standard. Along with a study with single-step GDH assay, we included a study that evaluated multi-step GDH assay when we could extract the separate GDH data from such study. Conference abstracts, short articles, and non-full articles were allowed.

#### Participants

Meta-analysis was conducted based on numbers of specimens but not on numbers of persons. Specimens from cases with a possible diagnosis of CDI, diarrheal stool, and liquid stood were preferred. When a study included formed specimens, we marked a high applicability concern for patient selection^22^. Human non-stool samples, animal stool samples, and food samples were excluded.

#### Index test

As an index test, we included any stool GDH assay including commercialized kit and in-house assays.

#### Reference test

The stool cell cytotoxicity neutralization assay (CCNA) and stool toxigenic culture (TC) were used as reference tests^8^. Other tests such as NAATs, and simple culture were not regarded as references in this study.

#### Outcome

First, we made a two by two contingency from the numbers of true positives/false negatives/false positives/true negatives presented in each original study. Then, we assessed the diagnostic odds ratio (DOR), and the area under the hierarchical SROC curve (AUC) to find the overall accuracy. The summary estimate of sensitivity, specificity, positive likelihood ratio (PLR), negative likelihood ratio (NLR), positive predictive value (PPV), and negative predictive value (NPV) were also assessed^16^.

### Literature search strategy

We had conducted a database search using PubMed, Embase, the Cochrane Library, and Web of Science on January 5^th^, 2016. Search formulas were presented in Supplementary Text 1.

References to previously published reviews and those of included original studies were hand-searched.

### Study selection

The two investigators independently conducted title/abstract screening after uploading a citation list into the software, Endnote X7 (THOMSON REUTERS, Philadelphia, USA). Articles that were not excluded by at least one investigator were passed for scrutiny. We scrutinized them by checking the full text independently. The final inclusion was determined after discussion to solve any discrepancies. Duplicate use of the same data was carefully excluded.

### Data extraction

The two investigators independently extracted data and input them into Microsoft Excel 2013. Then, the data extracted by the two investigators were crosschecked. Discrepancies were resolved by discussion between the two investigators.

### Quality assessment for bias and applicability

The two investigators independently evaluated each study. Seven domains of A Revised Tool for the Quality Assessment of Diagnostic Accuracy Studies (QUADAS-2) evaluation sheet were scored^22^. If the two investigators gave different scores, the discrepancies were resolved through discussion.

For the current systematic review, we assessed the quality using the following principles. Excluding patients for whom the authors had difficulty judging whether the patients had CDI or not was a reason for a high risk of patient selection bias. No description of consecutiveness and randomness was a reason for an unclear risk of patient selection. Including formed stool was a reason for a high patient selection applicability concern. Risk of bias for index and reference tests was generally not suspected because we can judge the results of GDH, CCNA, and TC unbiasedly. Bias in flow and timing was also not suspected because both index and reference tests were conducted on the same stool specimen.

A study without high risk of bias and high applicability concerns was regarded as a non-high-risk study.

### Statistical analysis and quantitative synthesis

#### Data synthesis

When, two GDH assays were compared to a reference test in a report, one assay was selected in the following order: Chek-60, Quik Chek, Culturette followed by Triage. This order was decided based on a number of studies that assessed each assay and a number of patients that were assessed for each assay. Data from two index assays in a study were independently used for index-test-based subgroup analysis. Similarly, when both CCNA and TC were used as references in a report, we chose CCNA as a reference test because recent study suggested that CCNA is more reliable than TC^23^. Data from two reference tests in a study were independently used for reference-test-based subgroup analysis.

We used both hierarchical SROC curves and bivariate models^16^,^17^,^18^,^19^. To assess the overall accuracy, we calculated the DOR using a DerSimonian-Laird random-model and the AUC using Holling’s proportional hazard models^24^,^25^. According to a criterion of Jones *et al*. AUC > 0.97, 0.93–0.96, 0.75–0.92, and 0.5–0.75 were interpreted as “excellent,” “very good,” “good,” and “reasonable,” respectively^26^. A paired forest plot, hierarchical SROC curve, and the summary estimate of the sensitivity and the specificity were obtained using the bivariate model 16. PLR and NLR were obtained from summary estimates of sensitivity and specificity. According to Grimes *et al*. PLR in the range of 2–5, 5–10, and >10 represent small, moderate, and large increases of probability when the test is positive. Similarly, NLR in the range of 0.2–0.5, 0.2–0.1, and <0.1 represent small, moderate, and large decreases of probability when the test is negative^27^. We also obtained PPV and NPV, which were calculated from summary estimates of sensitivity and specificity, as variables depending on pretest probability ranging from 0 to 100%.

As a sensitivity analysis, we conducted subgroup analysis including only non-high-risk studies and subgroup analysis based on reference tests. In addition, to compare the diagnostic accuracy, index-test-based subgroup analyses were carried out.

GRADE Evidence Profile table wad also presented^28^.

#### Heterogeneity

We used the I^2^ statistic to evaluate the heterogeneity of overall test accuracy among the studies: 0% meant no heterogeneity, 0% to 40% meant not important heterogeneity, 30% to 60% meant moderate heterogeneity, 50% to 90% meant substantial heterogeneity, 75% to 100% meant considerable heterogeneity^29^.

#### Software

A paired forest plot was made using Reviewing Manager ver. 5.3 (Cochrane Collaboration, Oxford, UK). The following commands of the “mada” package in the free software R were used: “madauni” for DOR, “phm” for AUC, and “reitsma” for the hierarchical SROC curve and a summary estimate for the sensitivity and the specificity^24^,^25^. GRADE evidence profile table was output from GRADE website^30^.

## Results

### Study search

Of 684 articles that met the preliminary criteria, 304, 213, and 125 were excluded through removal of duplication, title/abstract screening, and full-article scrutinization, respectively (Supplementary Figure 1). We finally found 42 eligible reports (Table 1, Supplementary Text 2). All the 42 reports used the cohort study approach and we found no case-control study. The 42 reports comprised 33 full-length articles, seven conference abstracts, a conference poster, and a letter article, all of which were written in English. Among the 42, 17 were from the USA, six were from Canada, six were from the UK, and most of the others were from developed countries. Seven reports described comparisons of two index tests and five reports described comparisons of reference CCNA and TC, thus, we eventually evaluated 54 cohorts.

**Figure 1:**
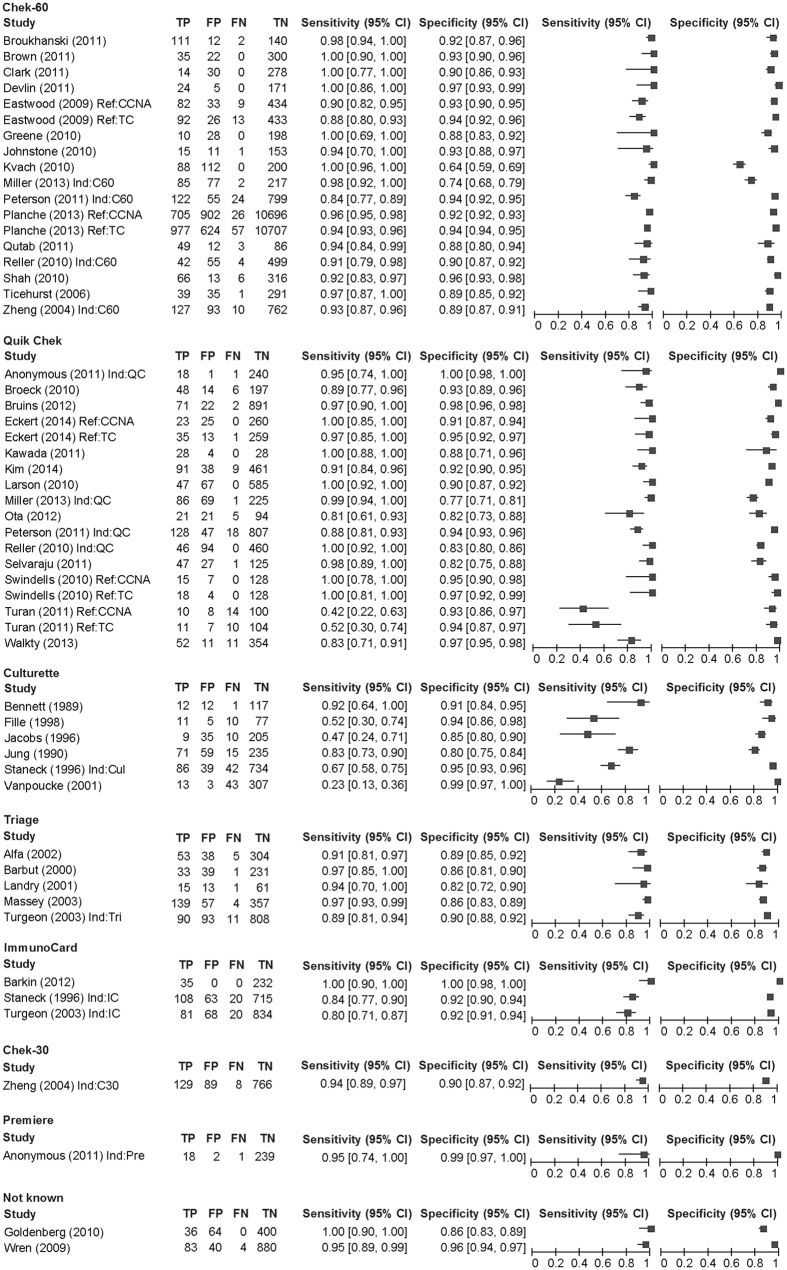
Paired forest plot. TP: true positive. FP: false positive. FN: false negative. TN: true negative. Ind: index test. Pre: Premiere. QC: Quik Chek. C60: Chek 60. Cul: Culturette. Tri: Triage. Ref: reference text. CCNA: cell cytotoxicity neutralization assay. TC: toxigenic culture.

As a reference test, 31 used CCNA and 23 used TC. As an index test, 18 used Chek-60, 18 used Quik Chek, six used the Culturette Brand Latex Test, and five used Triage. The comparison between the index and the reference in each cohort ranged from 60 to 12365 with a median of 373. The total number of comparisons was 47904, which consisted of 4946 reference positive comparisons and 42971 reference negative comparisons. Across the 54 cohorts, the sensitivity ranged from 0.23 to 1 with a median of 0.94 and the specificity ranged from 0.64 to 1 with a median of 0.92 (Fig. 1).

Among the 54 cohorts, 47 had high risk of flow and timing mostly due to duplicate use of multiple specimens from same patient. In addition, four had high risk of patient selection, three had high applicability concerns for patient selection, and one had high applicability concerns for the reference test (Supplementary Figure 2). Eventually six cohorts were classified as non-high-risk cohorts.

### Diagnostic accuracy across all index tests

**Figure 2:**
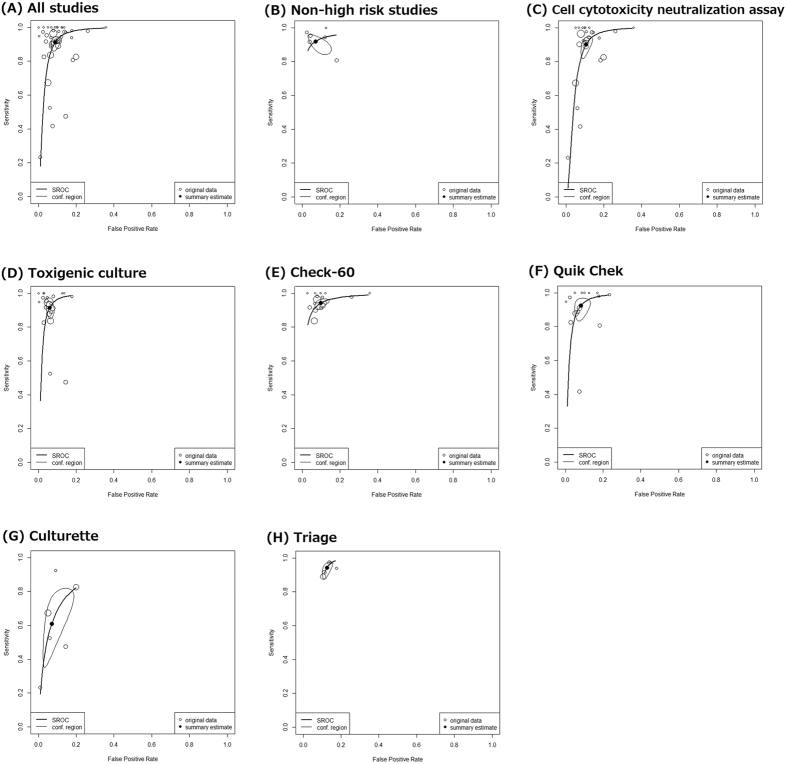
Hierarchical summary receiver-operator characteristic curves.

Using data from all 42 cohorts consisting of 3055 reference positive comparisons and 26188 reference negative comparisons, DOR was 115 (95% confidence interval (95% CI) 77–172, I^2^ = 12.0%) and AUC was 0.970 (95% CI 0.958–0.982) (Table 2, Fig. 2A). According to Jones’ criteria, the AUC of 0.970 meant excellent overall diagnostic accuracy^26^. According to the first sensitivity analysis using data from 6 non-high-risk cohorts with 2745 comparisons, DOR was 189 (95% CI 54–660, I^2^ = 0%) and AUC was 0.986 (95% CI 0.976–0.998) (Table 2, Fig. 2B). For the second sensitivity analysis based on CCNA, DOR was 80 (95% CI 50–131, I^2^ = 0%) and AUC was 0.956 (95% CI 0.927–0.987) (Table 2, Fig. 2C). For the third sensitivity analysis based on TC, DOR was 189 (95% CI 106–337, I^2^ = 27.2%) and AUC was 0.979 (95% CI 0.970–0.988) (Table 2, Fig. 2D).

According to the 42 cohorts, the summary estimate of sensitivity was 0.911 (95% CI 0.871–0.940) and the summary estimate of specificity was 0.912 (95% CI 0.892–0.928). These sensitivity and specificity estimates yielded PLR of 10.4 (95% CI 8.4–12.7) and NLR of 0.098 (95% CI 0.066–0.142). Based on Grimes’ criteria, these likelihood ratios suggested a large increase and decrease of probabilities, respectively^27^. PLR and NLR calculated in subgroup analysis focusing on non-high-risk cohorts and TC reference also suggested large increase and decrease of probabilities, respectively. However, PLR and NLR calculated in sensitivity analysis focusing on CCNA reference suggested a moderate increase and decrease of probabilities, respectively.

GRADE Evidence Profile was presented as Table 3. Supposing the protest pretest probability is in the range 15–25%^2^, among 1000 tested subjects, there are 137–228 true positives, 12–22 false negatives, 684–775 true negatives, and 66–75 false positives. PPV was 65–78% and NPV was 97–98%.

### Subgroup analysis based on index test

Check-60 was evaluated in 16 cohorts with 18737 comparisons. The DOR of 159 and AUC of 0.979 suggested excellent overall diagnostic accuracy. The sensitivity was 0.942 and the specificity was 0.901. The PLR of 9.5 and NLR of 0.064 suggested moderate increase and large decrease of likelihood ratio, respectively (Table 2, Figure 2E).

Quik Chek was evaluated in 15 cohorts with 6205 comparisons. The DOR of 152 and AUC of 0.980 also suggested excellent overall diagnostic accuracy. The sensitivity was 0.925 and the specificity was 0.918. The PLR of 11.3 and NLR of 0.082 suggested a large increase/decrease of the likelihood ratio (Table 2, Figure 2F).

Six cohorts evaluated the Culturette Latex agglutination test with 2151 comparisons. The AUC was 0.852 (95% CI 0.794–0.918) suggesting good overall diagnostic accuracy. The summary estimate of sensitivity of 0.610 was lower than those by Chek-60 and Quik Chek. The PLR was 8.6, which suggested a moderate increase of probability when the test is positive. The NLR was 0.420, which meant a small decrease of probability when the test is negative (Table 2, Figure 2G).

Five cohorts with 2353 comparisons assessed the diagnostic accuracy of Triage. Though excellent overall diagnostic accuracy was revealed by the AUC of 0.975, the specificity and PLR were lower than those for the other three assay kits (Table 2, Figure 2H).

## Discussion

To the best of our knowledge, this is the first meta-analysis to provide the summary estimate sensitivity and specificity of GDH detection for CDI. Our analysis showed that detecting GDH had excellent AUC and that test results from GDH greatly changed the probability of CDI. We believe that our result was robust for the careful study search, the use of hierarchical model, and low heterogeneity indicated by I^2^ < 30%. The quality-based subgroup analysis that replicated the results from all studies with any quality also support the robustness.

Reference-test-based sensitivity analysis revealed slightly discrepant results. When GDH assay was compared to reference test TC, the overall test accuracy was excellent. However, GDH assay seemed to have lower specificity when compared to reference test CCNA. Though both CCNA and TC are regarded as established standard examination for CDI, these two tests sometimes exhibit conflicting results. A large-scale prospective study by Planche *et al*. suggested that CCNA is a better reference test compared to TC because CCNA more accurately reflect mortality and CDI^23^. If we trust only the CCNA reference, the diagnostic accuracy of the GDH assay seems slightly degraded (Table 2, Figure 2C).

Index based subgroup analyses revealed that Chek-60 and Quik Chek, which were the most frequently evaluated kits, had the best performance. Although not supported by a sufficient number of studies, Triage seemed to lack specificity. The Culturette Brand Rapid Latex Test for CDI had clearly low diagnostic performance. Even though it detected GDH, this test was not designed for GDH. We have currently no reason to use the Culturette Brand Latex Test to detect GDH.

Once we assume the pretest probability was in the range 15–25%, PPV was 65–78% and NPV was 97–98%. While the GDH assay negative result is generally trustful, a positive GDH assay leads to wrong diagnosis for a third or a fourth of the tested population. Therefore, the currently used multi-step algorithm is a reasonable solution. In the medical resource abundant situation, NAATs can provide quick and accurate results for the second step. If use of NAATs is restricted, toxin detection is an alternative. However, toxin detection is not sensitive enough. Thus, we have to apply the NAATs as third step for GDH-positive toxin-negative specimens^31^. Even though some epidemiologic studies have suggested that CDI accounts for 15–25% of antibiotics-associated diarrhea, pretest probability should be judged by clinicians considering the patient’s clinical background and epidemiology in the area. Thus, the result of a GDH assay can be carefully interpreted.

To diagnose CDI in clinical practice, biochemical examinations that detect GDH, as well as toxin or nucleic acied of C. difficile in the stool of CDI-suspected patients are widely used. GDH is a metabolic enzyme that converts glutamate to α-ketoglutarate^8^,^9^,^10^,^11^. This enzyme commonly presents in many eukaryotes and microbes including C. difficile and other Clostridium species. To detect GDH in the stool, latex agglutination test was formerly used, whereas quantitative immunoassays are common these days. The key advantage of the enzyme immunoassays over the latex agglutination test is enhanced sensitivity due to quantitative evaluation using a standard curve. Moreover, the recently available lateral flow assay does not require a trained technician. Nowadays, we can obtain simple and accurate commercially-available enzyme immunoassay kits at low price though CCNA and TC are regarded as standard.

We need to comment on the limitations of our study. First, some of the included studies had high risk or high applicability concerns, therefore, we need to conduct sensitivity analysis excluding these studies. Second, subgroup analysis concerning the Culturette Latex test and Triage included a small number of studies; thus results were not sufficiently trustful. Third, the results were not consistent according to the reference tests. Thus, we provided GDH assay accuracies using two references separately. We believe these data are useful for future research. Fourth, recent advancement of PCR technique enables detection of a scarce load of microbes. PCR may be able to detect C. diff with higher sensitivity than culture though the culture is usually regarded as the gold standard. If we had used PCR as reference standard, the specificity would have been improved^32^.

In conclusion, we performed a systematic review and meta-analysis of the diagnostic test accuracy of detecting GDH for the diagnosis of CDI using a hierarchical model and a sufficient number of studies and comparisons. According to our analysis using 42 cohorts consisting of 29243 comparisons, the overall test accuracy was excellent, sensitivity was 0.911, specificity was 0.912, and the positive/negative results largely increased/decreased the probability of CDI. Suppose pretest probability was 15–25%, PPV was 65–78% and NPV was 97–98%.

## Additional Information

**How to cite this article**: Arimoto, J. *et al*. Diagnostic test accuracy of glutamate dehydrogenase for Clostridium difficile: Systematic review and meta-analysis. *Sci. Rep.* **6**, 29754; doi: 10.1038/srep29754 (2016).

## Supplementary Material

Supplementary Information

## Figures and Tables

**Table 1:** Characteristics of included cohorts.

Cohort name	Country	Study design	Report type	Specimen type	Facility	Reference test	Index test	Comparisons	Non-high-risk
Alfa (2002)	Canada	pCohort	Full A	s/o CDI	A tertiary hospital	CCNA	Triage	400	Yes
Anonymous (2011) Ind:Pre		pCohort	C Abst	Liquid stool		TC	Premiere	260	Yes
Anonymous (2011) Ind:QC		pCohort	C Abst	Liquid stool		TC	Quik Chek	260	Yes
Barbut (2000)	France	Cohort	Full A	Diarrhea	Hospitals	CCNA	Triage	304	Yes
Barkin (2012)	USA	rCohort	Full A	s/o CDI	A community teaching hospital	TC	ImmunoCard	267	Yes
Bennett (1989)	USA	Cohort	Full A	Diarrhea	Nursing homes	CCNA	Culturette	142	Yes
Broeck (2010)	Belgium	Cohort	C Post	Diarrhea	A university hospital	TC	Quik Chek	265	Yes
Broukhanski (2011)	Canada		C Abst			TC	Chek-60	265	Yes
Brown (2011)	USA	Cohort	Full A	Liquid stool	A test center	CCNA	Chek-60	357	Yes
Bruins (2012)	Netherlands	Cohort	Full A	s/o CDI	A laboratory	TC	Quik Chek	986	Yes
Clark (2011)	UK	pCohort	Letter	Diarrhea	A hospital	CCNA	Chek-60	322	Yes
Devlin (2011)	Canada	Cohort	C Abst			TC	Chek-60	200	Yes
Eastwood (2009) Ref:CCNA	UK	pCohort	Full A	Diarrhea	Teaching hospitals	CCNA	Chek-60	558	Yes
Eastwood (2009) Ref:TC	UK	pCohort	Full A	Diarrhea	Teaching hospitals	TC	Chek-60	564	Yes
Eckert (2014) Ref:CCNA	France	pCohort	Full A	s/o CDI, diarrhea	A test center	CCNA	Quik Chek	308	Yes
Eckert (2014) Ref:TC	France	pCohort	Full A	s/o CDI, diarrhea	A test center	TC	Quik Chek	308	Yes
Fille (1998)	Austria	Cohort	Full A	s/o CDI	A laboratory	CCNA	Culturette	103	No
Goldenberg (2010)	UK	Cohort	Full A	Diarrhea	A hospital	TC	Chek	500	Yes
Greene (2010)	USA		C Abst			CCNA	Chek-60	236	No
Jacobs (1996)	Israel	Cohort	Full A	Diarrhea	A teaching hospital	TC	Culturette	259	Yes
Johnstone (2010)	Canada		C Abst			TC	Chek-60	180	Yes
Jung (1990)	Sweden	Cohort	Full A	s/o CDI		CCNA	Culturette	380	Yes
Kawada (2011)	Japan	pCohort	Full A	s/o CDI	A hospital	TC	Quik Chek	60	Yes
Kim (2014)	Korea	Cohort	Full A	s/o CDI, loose stool	A tertiary teaching hospital	TC	Quik Chek	599	Yes
Kvach (2010)	USA	Cohort	Full A	Liquid stool/Diarrhea	A hospital	CCNA	Chek-60	400	Yes
Landry (2001)	USA	pCohort	Full A		A hospital	CCNA	Triage	90	No
Larson (2010)	USA	Cohort	Full A	Soft/liquid stool	A medical center	CCNA	Quik Chek	699	Yes
Massey (2003)	Canada	Cohort	Full A	s/o CDI		CCNA	Triage	557	Yes
Miller (2013) Ind:C60	USA	Cohort	Full A	s/o CDI, liquid stool	A university hospital	CCNA	Chek-60	381	Yes
Miller (2013) Ind:QC	USA	Cohort	Full A	s/o CDI, liquid stool	A university hospital	CCNA	Quik Chek	381	Yes
Ota (2012)	USA	pCohort	Full A	Liquid stool	A children hospital	CCNA	Quik Chek	141	Yes
Peterson (2011) Ind:C60	USA	Cohort	Full A	s/o CDI	university laboratory	TC	Chek-60	1000	Yes
Peterson (2011) Ind:QC	USA	Cohort	Full A	s/o CDI	university laboratory	TC	Quik Chek	1000	Yes
Planche (2013) Ref:CCNA	UK	pCohort	Full A	Bristol 5–7	Teaching hospitals	CCNA	Chek-60	12329	Yes
Planche (2013) Ref:TC	UK	pCohort	Full A	Bristol 5–7	Teaching hospitals	TC	Chek-60	12365	Yes
Qutab (2011)	Saudi Arabia	Cohort	Full A	s/o CDI		CCNA	Chek-60	150	Yes
Reller (2010) Ind:C60	USA	Cohort	Full A			CCNA	Chek-60	600	Yes
Reller (2010) Ind:QC	USA	Cohort	Full A			CCNA	Quik Chek	600	Yes
Selvaraju (2011)	USA	Cohort	Full A	Liquid/soft/formed stool		TC	Quik Chek	200	No
Shah (2010)	USA	Cohort	C Abst			TC	Chek-60	401	Yes
Staneck (1996) Ind:Cul	USA	rCohort	Full A	AAD	University hospitals	CCNA	Culturette	901	No
Staneck (1996) Ind:IC	USA	rCohort	Full A	AAD	University hospitals	CCNA	ImmunoCard	906	No
Swindells (2010) Ref:CCNA	UK	Cohort	Full A	Diarrhea, >65yo		CCNA	Quik Chek	150	Yes
Swindells (2010) Ref:TC	UK	Cohort	Full A	Diarrhea, >66yo		TC	Quik Chek	150	Yes
Ticehurst (2006)	USA	Cohort	Full A		Teaching hospitals	CCNA	Chek-60	366	Yes
Turan (2011) Ref:CCNA	Turkey	Cohort	C Abst	s/o CDI		CCNA	Quik Chek	132	Yes
Turan (2011) Ref:TC	Turkey	Cohort	C Abst	s/o CDI		TC	Quik Chek	132	Yes
Turgeon (2003) Ind:IC	USA	Cohort	Full A	Stool with any consistency	Hosptals	CCNA	ImmunoCard	1003	No
Turgeon (2003) Ind:Tri	USA	Cohort	Full A	Stool with any consistency	Multicenter	CCNA	Triage	1002	No
Vanpoucke (2001)	Belgium	Cohort	Full A	s/o CDI, liquid/semi-liquid stool	A university hospital	CCNA	Culturette	366	Yes
Walkty (2013)	Canada	Cohort	Full A	Diarrhea	A hospital and laboratories	TC	Quik Chek	428	Yes
Wren (2009)	UK	Cohort	Full A	s/o CDI		TC		1007	Yes
Zheng (2004) Ind:C30	USA	Cohort	Full A		Test centers	CCNA	Chek-30	992	Yes
Zheng (2004) Ind:C60	USA	Cohort	Full A		Test centers	CCNA	Chek-60	992	Yes

When a report compared an index test with two reference tests or when a report compared two index test with a reference, we regarded such a report as two independent study. <Cohort name > Ref: Reference test. Ind: Index test. Pre: Premiere. QC: Quik Chek. C60: Chek-60. IC: ImmunoCard. Cul: Culturette. Tri: Triage. C30: Chek-30. <Study design > pCohort: prospective cohort. rCohort: retrospective cohort. <Report type > Full A: full-length article. C Abst: conference abstract. C Post: conference poster. <Specimen type > s/o CDI: suspected of C. difficiele. AAD: Antibiotics-associated diarrhea < Reference test> CCNA: Cell cytotoxicity neutralization assay. TC: Toxigenic culture.

**Table 2:** Summary of results

Reference	(A)	(B)	(C)	(D)	(E)	(F)	(G)	(H)
	Any	Any	CCNA	TC	Any	Any	Any	Any
Index	Any	Any	Any	Any	Chek-60	Quik Chek	Culturette	Triage
Non-high-risk	Any	Yes	Any	Any	Any	Any	Any	Any
N	42	6	26	21	16	15	6	5
n	29243	2745	22366	20396	18737	6209	2151	2353
DOR	115	189	80	189	159	152	22	97
(95%CI)	(77–172)	(54–660)	(50–131)	(106–337)	(104–243)	(75–308)	(11–43)	(61–154)
I^2^	12.0%	0%	0%	27.2%	0%	13.0%	9.5%	0%
AUC	0.970	0.986	0.956	0.979	0.979	0.980	0.852	0.975
(95%CI)	(0.958–0.982)	(0.976–0.998)	(0.927–0.987)	(0.970–0.988)	(0.970–0.989)	(0.968–0.992)	(0.794–0.918)	(0.959–0.991)
Sensitivity	0.911	0.919	0.901	0.914	0.942	0.925	0.610	0.943
(95%CI)	(0.871–0.940)	(0.861–0.955)	(0.838–0.941)	(0.865–0.947)	(0.913–0.962)	(0.857–0.962)	(0.600–0.786)	(0.891–0.971)
Specificity	0.912	0.929	0.894	0.941	0.901	0.918	0.929	0.874
(95%CI)	(0.892–0.928)	(0.867–0.964)	(0.867–0.916)	(0.922–0.955)	(0.867–0.927)	(0.879–0.945)	(0.843–0.969)	(0.851–0.895)
PLR	10.4	12.9	8.5	15.5	9.5	11.3	8.6	7.5
(95%CI)	(8.4–12.7)	(6.8–25.2)	(6.7–10.8)	(11.7–20.4)	(7.1–12.9)	(7.6–16.8)	(3.8–20.1)	(6.2–8.9)
NLR	0.098	0.087	0.111	0.091	0.064	0.082	0.420	0.065
(95%CI)	(0.066–0.142)	(0.049–0.152)	(0.066–0.181)	(0.057–0.144)	(0.042–0.097)	(0.041–0.156)	(0.312–0.548)	(0.033–0.125)

N: number of cohorts. n: number of comparisons. SROC: summary receiver operating characteristics. AUC: area under hierarchical summary receiver operating characteristics curve. PLR: positive likelihood ratio. NLR: negative likelihood ratio. 95% CI: 95% confidence interval.

**Table 3:**
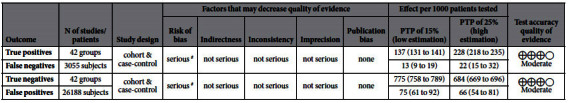
GRADE evidence profile for diagnostic test accuracy by detecting glutamate dehydrogenase assay for Clostridium difficile infection (CDI).

This table was based on following statistics: sensitivity 0.911 (95% CI: 0.871 to 0.940), specificity 0.912 (95% CI: 0.892 to 0.928), prevalence 15–25%. PTP: pre-test probability. ^#^Most studies had high risk for “flow and timing”.
