# Supplementary material for: Diagnostic test accuracy of glutamate dehydrogenase for Clostridium difficile: Systematic review and meta-analysis
Source: Sci Rep. 2016 Jul 15;6:29754. doi: 10.1038/srep29754 (PMC4945925; doi:10.1038/srep29754)
Supplement: Supplementary Information [file srep29754-s1.doc]

**Diagnostic test accuracy of glutamate dehydrogenase for Clostridium difficile:**

**Systematic review and meta-analysis**

**<<Supplementary File>>**

**Authors**

^1)^Jun Arimoto, ^2)^Nobuyuki Horita, ^1)^Shingo Kato, ^1)^Akiko Fuyuki, ^1)^Takuma Higurashi, ^1)^Hidenori Ohkubo, ^1)^Hiroki Endo, ^1)^Takashi, Nonaka, ^2)^Takeshi Kaneko, ^1)^Atsushi Nakajima,

^1)^Department of Gastroenterology and Hepatology, Yokohama City University School of Medicine, Yokohama, Japan.

^2)^Department of Pulmonology, Yokohama City University Graduate School of Medicine, Yokohama, Japan.

**Supplementary Text 1. Search formulas.**

*PubMed:*

(difficile OR "Clostridium diff" OR "C. diff" OR "C diff" OR "pseudomembranous colitis" OR PMC) AND (sensitivity OR specificity OR "predictive value" OR "likelihood" OR "true positive" OR "true negative" OR "false positive" OR "false negative" OR diagnostic OR diagnosis) AND (Culturette OR "common antigen" OR GDH OR (glutamate dehydrogenase) OR CHEK-60 OR TL-GDH OR ImmunoCard OR (Quik Chek Complete) OR (Triage Panel) OR (Vidas Panel)).

*EMBASE:*

('pseudomembranous colitis' OR 'difficile' OR 'clostridium diff' OR 'pseudomembranous colitis'/exp) AND (sensitivity OR specificity OR 'predictive value' OR 'likelihood' OR 'true positive' OR 'true negative' OR 'false positive' OR 'false negative' OR diagnostic OR diagnosis) AND (Culturette:ab,ti OR 'common antigen':ab,ti OR gdh OR 'glutamate dehydrogenase' OR 'glutamate dehydrogenase'/exp OR 'chek 60' OR 'tl gdh' OR immunocard OR (quik AND chek AND complete) OR (triage AND panel) OR (vidas AND panel)).

*Cochrane Library, title/abstract/keyword search without limitation:*

(difficile OR "Clostridium diff" OR "C. diff" OR "C diff" OR "pseudomembranous colitis" OR PMC) AND (sensitivity OR specificity OR "predictive value" OR "likelihood" OR "true positive" OR "true negative" OR "false positive" OR "false negative" OR diagnostic OR diagnosis) AND (Culturette OR "common antigen" OR GDH OR (glutamate dehydrogenase) OR CHEK-60 OR TL-GDH OR ImmunoCard OR (Quik Chek Complete) OR (Triage Panel) OR (Vidas Panel)).

*Web of Science Core collection advanced search without limitation:*

TS=(difficile OR "Clostridium diff" OR "C. diff" OR "C diff" OR "pseudomembranous colitis" OR PMC) AND TS=(sensitivity OR specificity OR "predictive value" OR "likelihood" OR "true positive" OR "true negative" OR "false positive" OR "false negative" OR diagnostic OR diagnosis) AND TS=(Culturette OR "common antigen" OR GDH OR (glutamate dehydrogenase) OR CHEK-60 OR TL-GDH OR ImmunoCard OR (Quik Chek Complete) OR (Triage Panel) OR (Vidas Panel)).

**Supplementary Text 2. List of included studies.**

1 Alfa, M. J., Swan, B., VanDekerkhove, B., Pang, P. & Harding, G. K. The diagnosis of Clostridium difficile-associated diarrhea: comparison of Triage C. difficile panel, EIA for Tox A/B and cytotoxin assays. *Diagn Microbiol Infect Dis* **43**, 257-263 (2002).

2 Annonymous. Evaluation of a novel loop-mediated amplification system (illumi-pro 10 meridian bioscience incs), glutamate dehydrogenase-GDH (meridian and Techlab) and a rapid immunoassay-Tox A/B QUIK CHEK (Techlab) for the detection of C. Difficile toxin. *J Infect* **63**,e95-e96 (2011).

3 Barbut, F. *et al.* Usefulness of simultaneous detection of toxin A and glutamate dehydrogenase for the diagnosis of Clostridium difficile-associated diseases. *Eur J Clin Microbiol Infect Dis* **19**, 481-484 (2000).

4 Barkin, J. A. *et al.* Superiority of the DNA amplification assay for the diagnosis of C. difficile infection: a clinical comparison of fecal tests. *Dig Dis Sci* **57**, 2592-2599, doi:10.1007/s10620-012-2200-x (2012).

5 Bennett, R. G. *et al.* Evaluation of a latex agglutination test for Clostridium difficile in two nursing home outbreaks. *J Clin Microbiol* **27**, 889-893 (1989).

6 Van Broeck, J., Hubert, C., Vast, M., & Delmée, M., A two-step algorithm for the diagnosis of Clostridium difficile infection: Screening with a rapid immunoassay for the detection of glutamatedehydrogenase and toxins A and B followed by a real-time PCR for C. difficile. *Clin Microbiol Infect* **16**, S167-S168 (2010).

7 Broukhanski, G., Maki, A., Peralta, A., & Pillai, D., R. Evaluation of Techlab QUICK CHEK™-60 gluta matedehydrogenase test for detection of clostridium difficile in clinical specimens. *Can J Infect Dis Med Microbiol* **22**, 24A (2011).

8 Brown, N. A., Lebar, W. D., Young, C. L., Hankerd, R. E. & Newton, D. W. Diagnosis of Clostridium difficile infection: comparison of four methods on specimens collected in Cary-Blair transport medium and tcdB PCR on fresh versus frozen samples. *Infect Dis Rep* **3**, e5, doi:10.4081/idr.2011.e5 (2011).

9 Bruins, M. J. *et al.* Evaluation of three enzyme immunoassays and a loop-mediated isothermal amplification test for the laboratory diagnosis of Clostridium difficile infection. *Eur J Clin Microbiol Infect Dis* **31**, 3035-3039, doi:10.1007/s10096-012-1658-y (2012).

10 Clark, N. S., Doughton, M. J., Karas, J. A. & Enoch, D. A. Two-step algorithm for the detection of Clostridium difficile from stool samples. *J Hosp Infect* **79**, 95-96; author reply 96-97, doi:10.1016/j.jhin.2011.04.002 (2011).

11 Devlin, H., R., Jackson, C., Pangan, O., Broukhanski, G., & Pillai, D., R. Rapid and accurate diagnosis of clostridium difficile infection. *Can J Infect Dis Med Microbiol* **22**, 24A (2011).

12 Eastwood, K., Else, P., Charlett, A. & Wilcox, M. Comparison of nine commercially available Clostridium difficile toxin detection assays, a real-time PCR assay for C. difficile tcdB, and a glutamate dehydrogenase detection assay to cytotoxin testing and cytotoxigenic culture methods. *J Clin Microbiol* **47**, 3211-3217, doi:10.1128/JCM.01082-09 (2009).

13 Eckert, C., Holscher, E., Petit, A., Lalande, V. & Barbut, F. Molecular test based on isothermal helicase-dependent amplification for detection of the Clostridium difficile toxin A gene. *J Clin Microbiol* **52**, 2386-2389, doi:10.1128/JCM.00353-14 (2014).

14 Fille, M., Larcher, C., Dierich, M. P. & Allerberger, F. Evaluation of four methods for detection of Clostridium difficile or C. difficile toxin: cytotoxin assay, culture, latex agglutination, and a new rapid immunoassay (C. difficile toxin A test). *Z Gastroenterol* **36**, 143-149 (1998).

15 Goldenberg, S. D., Cliff, P. R., Smith, S., Milner, M. & French, G. L. Two-step glutamate dehydrogenase antigen real-time polymerase chain reaction assay for detection of toxigenic Clostridium difficile. *J Hosp Infect* **74**, 48-54, doi:10.1016/j.jhin.2009.08.014 (2010).

16 Greene, W. Evaluation of the C. difficile TOXIN A/B IITM, C. DIFF CHEK-60TM, and PREMIER TOXIN A& BTM with the cytotoxin assay for the detection of Clostridium difficile GDH antigen and toxins A and B in feces. *Int J Infect Dis* **14**,e369 (2010).

17 Jacobs, J. *et al.* Comparison of four laboratory tests for diagnosis of Clostridium difficile-associated diarrhea. *Eur J Clin Microbiol Infect Dis* **15**, 561-566 (1996).

18 Johnstone, D., Toye, B., & Desjardins, M. Laboratory diagnosis of C. difficile (CD) infection (CDI): Techlab C. difficile Tox A/B II EIA (Tox AB), Techlab C. difficile chek-60 EIA (GDH), becton dickinson geneohm cdiff PCR (BD-PCR), and the prodesse progastro CD PCRs (PG-PCR) compared to toxigenic culture. *Can J Infect Dis Med Microbiol*,**21**,5A (2010).

19 Jung, K. & Aronsson, B. Rapid diagnosis of Clostridium difficile-associated diarrhoea using a latex agglutination test. *APMIS* **98**, 652-654 (1990).

20 Kawada, M. *et al.* Evaluation of a simultaneous detection kit for the glutamate dehydrogenase antigen and toxin A/B in feces for diagnosis of Clostridium difficile infection. *J Infect Chemother* **17**, 807-811, doi:10.1007/s10156-011-0267-5 (2011).

21 Kim, H., Kim, W. H., Kim, M., Jeong, S. H. & Lee, K. Evaluation of a rapid membrane enzyme immunoassay for the simultaneous detection of glutamate dehydrogenase and toxin for the diagnosis of Clostridium difficile infection. *Ann Lab Med* **34**, 235-239, doi:10.3343/alm.2014.34.3.235 (2014).

22 Kvach, E. J., Ferguson, D., Riska, P. F. & Landry, M. L. Comparison of BD GeneOhm Cdiff real-time PCR assay with a two-step algorithm and a toxin A/B enzyme-linked immunosorbent assay for diagnosis of toxigenic Clostridium difficile infection. *J Clin Microbiol* **48**, 109-114, doi:10.1128/JCM.01630-09 (2010).

23 Landry, M. L., Topal, J., Ferguson, D., Giudetti, D. & Tang, Y. Evaluation of biosite triage Clostridium difficile panel for rapid detection of Clostridium difficile in stool samples. *J Clin Microbiol* **39**, 1855-1858, doi:10.1128/JCM.39.5.1855-1858.2001 (2001).

24 Larson, A. M., Fung, A. M. & Fang, F. C. Evaluation of tcdB real-time PCR in a three-step diagnostic algorithm for detection of toxigenic Clostridium difficile. *J Clin Microbiol* **48**, 124-130, doi:10.1128/JCM.00734-09 (2010).

25 Massey, V. *et al.* Clinical usefulness of components of the Triage immunoassay, enzyme immunoassay for toxins A and B, and cytotoxin B tissue culture assay for the diagnosis of Clostridium difficile diarrhea. *Am J Clin Pathol* **119**, 45-49, doi:10.1309/U8AT-L52Q-60XY-AVX6 (2003).

26 Miller, S., Wiita, A., Wright, C., Reyes, H., & Liu, C. Evaluation of glutamate dehydrogenase immunoassay screening with toxin confirmation for the diagnosis of clostridium difficile infection. Lab Med. 2013;44:e65-e71.

27 Ota, K. V. & McGowan, K. L. Clostridium difficile testing algorithms using glutamate dehydrogenase antigen and C. difficile toxin enzyme immunoassays with C. difficile nucleic acid amplification testing increase diagnostic yield in a tertiary pediatric population. *J Clin Microbiol* **50**, 1185-1188, doi:10.1128/JCM.05620-11 (2012).

28 Peterson, L. R. *et al.* Laboratory testing for Clostridium difficile infection: light at the end of the tunnel. *Am J Clin Pathol* **136**, 372-380, doi:10.1309/AJCPTP5XKRSNXVIL (2011).

29 Planche, T. D. *et al.* Differences in outcome according to Clostridium difficile testing method: a prospective multicentre diagnostic validation study of C difficile infection. *Lancet Infect Dis* **13**, 936-945, doi:10.1016/S1473-3099(13)70200-7 (2013).

30 Qutub, M. O., AlBaz, N., Hawken, P. & Anoos, A. Comparison between the two-step and the three-step algorithms for the detection of toxigenic Clostridium difficile. *Indian J Med Microbiol* **29**, 293-296, doi:10.4103/0255-0857.83916 (2011).

31 Reller, M. E., Alcabasa, R. C., Lema, C. A. & Carroll, K. C. Comparison of two rapid assays for Clostridium difficile Common antigen and a C difficile toxin A/B assay with the cell culture neutralization assay. *Am J Clin Pathol* **133**, 107-109, doi:10.1309/AJCPO3QWOU8CYGEU (2010).

32 Selvaraju, S. B. *et al.* Detection of toxigenic Clostridium difficile in pediatric stool samples: an evaluation of Quik Check Complete Antigen assay, BD GeneOhm Cdiff PCR, and ProGastro Cd PCR assays. *Diagn Microbiol Infect Dis* **71**, 224-229, doi:10.1016/j.diagmicrobio.2011.07.015 (2011).

33 Shah, M., Stojan, G., Crist, A., & Bell, T. Clostridium difficile infection: Comparison of available laboratory diagnostic methods and clinical correlations. *Am J Gastroenterol* **105**, S132-S133 (2010).

34 Staneck, J. L. *et al.* Multicenter evaluation of four methods for Clostridium difficile detection: ImmunoCard C. difficile, cytotoxin assay, culture, and latex agglutination. *J Clin Microbiol* **34**, 2718-2721 (1996).

35 Swindells, J., Brenwald, N., Reading, N. & Oppenheim, B. Evaluation of diagnostic tests for Clostridium difficile infection. *J Clin Microbiol* **48**, 606-608, doi:10.1128/JCM.01579-09 (2010).

36 Ticehurst, J. R. *et al.* Effective detection of toxigenic Clostridium difficile by a two-step algorithm including tests for antigen and cytotoxin. *J Clin Microbiol* **44**, 1145-1149, doi:10.1128/JCM.44.3.1145-1149.2006 (2006).

37 Turan, M. et al. Evaluation of a real-time PCR assay and conventional methods for the detection of toxigenic Clostridium difficile in stool samples. *Clin Microbiol Infect*, **17**, S582-S583 (2011).

38 Turgeon, D. K. *et al.* Six rapid tests for direct detection of Clostridium difficile and its toxins in fecal samples compared with the fibroblast cytotoxicity assay. *J Clin Microbiol* **41**, 667-670 (2003).

39 Vanpoucke, H., De Baere, T., Claeys, G., Vaneechoutte, M. & Verschraegen, G. Evaluation of six commercial assays for the rapid detection of Clostridium difficile toxin and/or antigen in stool specimens. *Clin Microbiol Infect* **7**, 55-64 (2001).

40 Walkty, A. *et al.* Evaluation of an algorithmic approach in comparison with the Illumigene assay for laboratory diagnosis of Clostridium difficile infection. *J Clin Microbiol* **51**, 1152-1157, doi:10.1128/JCM.03203-12 (2013).

41 Wren, M.W., Sivapalan, M., Kinson, R., & Shetty, N.R. Laboratory diagnosis of clostridium difficile infection. An evaluation of tests for faecal toxin, glutamate dehydrogenase, lactoferrin and toxigenic culture in the diagnostic laboratory. *Br J Biomed Sci* **66**, 1-5 (2009).

42 Zheng, L. *et al.* Multicenter evaluation of a new screening test that detects Clostridium difficile in fecal specimens. *J Clin Microbiol* **42**, 3837-3840, doi:10.1128/JCM.42.8.3837-3840.2004 (2004).

**Supplementary Figure 1. The Preferred Reporting Items for Systematic Reviews and Meta- Analyses (PRISMA) flow diagram.**

**
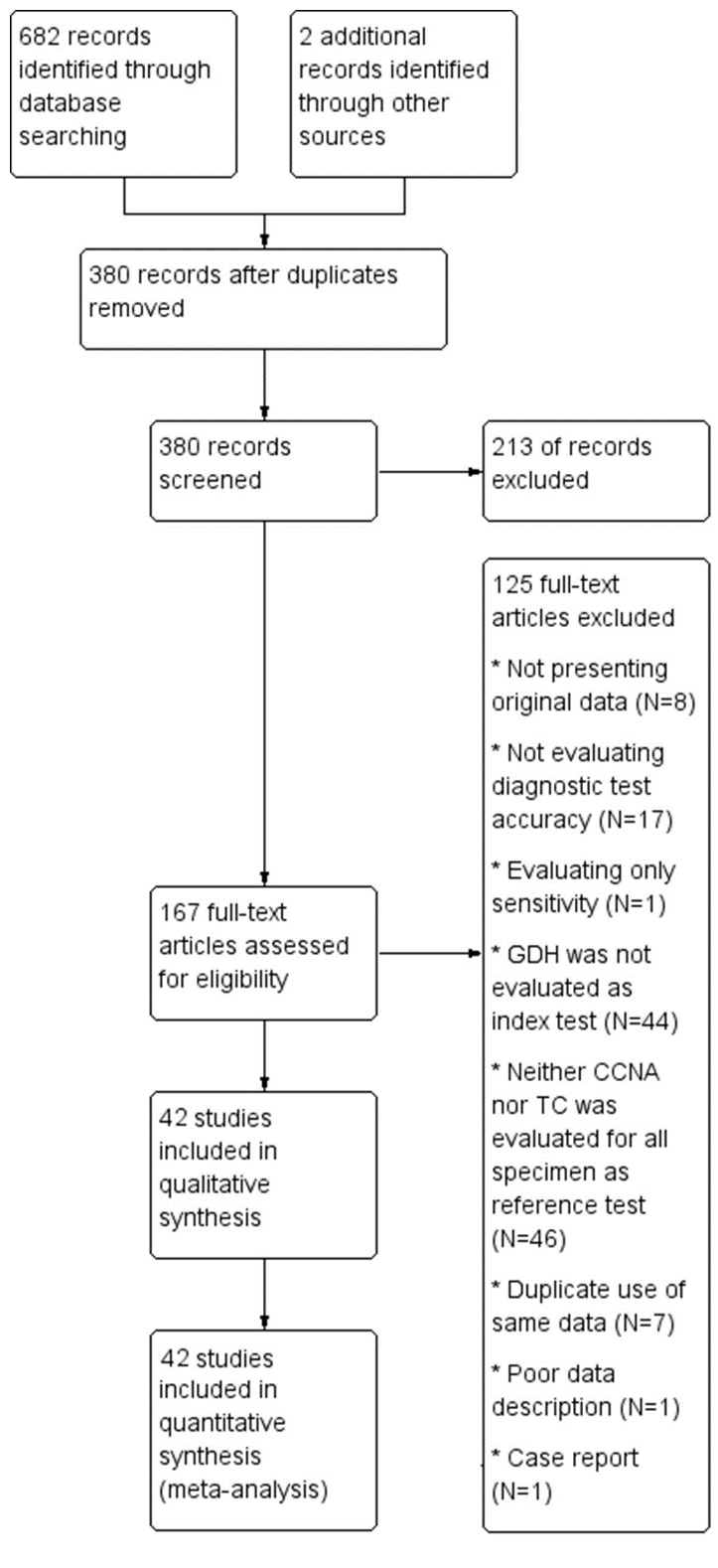
**


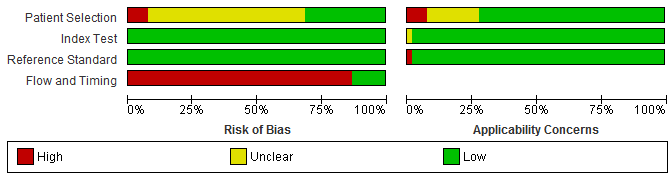
**Supplementary Figure 2. Risk of bias and applicability concerns graph**
